# Supplementary material for: MetaRibo-Seq measures translation in microbiomes
Source: Nat Commun. 2020 Jun 29;11:3268. doi: 10.1038/s41467-020-17081-z (PMC7324362; doi:10.1038/s41467-020-17081-z)
Supplement: Supplementary file 10 — Supplementary Data 7 [file 41467_2020_17081_MOESM10_ESM.zip › File2/Confidence_High_Taxonomy/109156_out.krona.html]

Javascript must be enabled to view this page.

members
magnitude
magnitudeUnassigned
count
unassigned
taxon
rank

109156\_out

58

2
58
superkingdom

phylum
58
1239

58
class
186801

58
order
186802

58
186803
family

genus
58
841

58
species
166486

SRS013158\_contig\_number\_3482SRS013951\_contig\_number\_16216SRS014287\_contig\_number\_16817SRS014613\_contig\_number\_21517SRS014855\_contig\_number\_17636SRS015190\_contig\_number\_16055SRS015264\_contig\_number\_17981SRS015579\_contig\_number\_21037SRS015854\_contig\_number\_18755SRS015890\_contig\_number\_26212SRS015960\_contig\_number\_20360SRS016335\_contig\_number\_38594SRS017821\_contig\_number\_16273SRS018984\_contig\_number\_19633SRS019397\_contig\_number\_2383SRS019787\_contig\_number\_contig-100\_2080.34448SRS022137\_contig\_number\_20345SRS024435\_contig\_number\_contig-100\_1051.253086SRS024549\_contig\_number\_contig-100\_1336.42742SRS043001\_contig\_number\_contig-100\_1565.70242SRS043411\_contig\_number\_18244SRS043701\_contig\_number\_contig-100\_1633.116276SRS045739\_contig\_number\_9639SRS046068\_contig\_number\_contig-100\_2609.2610SRS049959\_contig\_number\_11593SRS050422\_contig\_number\_36963SRS052027\_contig\_number\_17375SRS053335\_contig\_number\_17156SRS054956\_contig\_number\_10667SRS055017\_contig\_number\_17130SRS056519\_contig\_number\_17400SRS075716\_contig\_number\_943SRS076756\_contig\_number\_21072SRS077454\_contig\_number\_17047SRS077552\_contig\_number\_6341SRS077641\_contig\_number\_16678SRS098514\_contig\_number\_360SRS098881\_contig\_number\_26434SRS104084\_contig\_number\_11097SRS1041130\_contig\_number\_1814SRS104975\_contig\_number\_14282SRS105153\_contig\_number\_34909SRS1055034\_contig\_number\_12097SRS1055067\_contig\_number\_9270SRS142503\_contig\_number\_21019SRS142781\_contig\_number\_11520SRS143070\_contig\_number\_10991SRS143417\_contig\_number\_11195SRS143598\_contig\_number\_23825SRS143722\_contig\_number\_16460SRS143780\_contig\_number\_20635SRS144537\_contig\_number\_contig-100\_6641.175485SRS147919\_contig\_number\_14013SRS148091\_contig\_number\_4655SRS148874\_contig\_number\_contig-100\_2288.2289SRS149181\_contig\_number\_16882SRS893378\_contig\_number\_11956SRS893380\_contig\_number\_contig-100\_1604.1605
